# Computational insights into survival durations and prehospital interventions in accidental cold-water immersion: A comprehensive analysis of fresh and saltwater temperatures

**DOI:** 10.1016/j.heliyon.2024.e33022

**Published:** 2024-06-13

**Authors:** Mohammad Junaid, Md Mahmud-Or-Rashid

**Affiliations:** Department of Mechanical Engineering, Shahjalal University of Science and Technology, Sylhet, 3114, Bangladesh

**Keywords:** Whole body model, Bio-heat, Coldwater immersion, Accidental hypothermia, Rewarming, Rectal temperature, Titanic accident, Finite element, Prehospital hypothermia treatments

## Abstract

This study examines the complex relationship between scenarios of cold-water immersion, survival durations, and prehospital interventions. It utilizes computational modeling methods to shed light on how different water temperatures affect individuals facing accidental cold-water immersion incidents. The analysis reveals significant variations in survival times based on water temperature. For example, subjects immersed in water at temperatures of 5 °C, 2 °C, and 0 °C had average survival times of 136, 113, and 100 min, respectively, under stable conditions. In flowing water at the same temperatures, survival times decreased to 119, 92, and 81 min, indicating the impact of water movement on cooling rates and survival durations. Likewise, individuals immersed in saltwater at temperatures of 5 °C, 2 °C, 0 °C, and −2 °C showed average survival times of 111, 88, 80, and 66 min, respectively, in static conditions. In flowing saltwater at the same temperatures, survival times decreased to 98, 74, 68, and 57 min, highlighting the influence of water flow on cooling rates and survival durations. A comparison between immersion in pure water and saltwater at 2 °C revealed survival times of 113 and 88 min under stable conditions and 92 and 74 min under dynamic conditions, emphasizing the role of water composition in survival outcomes. The study also challenges the notion that the demise of the Titanic's passengers and crew resulted from hypothermia, asserting instead that severe thermal shock was the primary cause. These numerical findings underscore the importance of considering water temperature, flow dynamics, and prompt medical responses in cold-water emergencies to enhance survival prospects. The study identifies water within the range of 41–43 °C as the most effective active external rewarming fluid for critical hypothermal conditions. By quantifying the impact of these variables on survival times, the study provides data-driven recommendations to improve emergency protocols and outcomes for individuals facing cold-water immersion incidents.

## 1 Introduction

Examining how the human body responds to different thermal stress conditions presents a complex challenge. The objective of using a human body model is to evaluate both the steady and dynamic reactions of the human body under various environmental conditions and during different physical activities. To achieve an accurate representation of the human thermoregulatory system and its interaction with the environment, realistic modeling techniques are necessary. The foundational framework for determining the temperature distribution within a human limb in a steady state was introduced by Pennes in 1948 [1], where the limb was initially conceptualized as a cylindrical structure. Pennes' model incorporated important physiological complexities, including factors such as variations in metabolic heat generation, blood perfusion, radial heat conduction, and heat loss mechanisms such as convection, radiation, and evaporation from the cylindrical surface. In this study, Pennes' bioheat equation uses blood temperature as an initial value during the onset of transient conditions. Importantly, this equation can be extended to different regions of the human body by representing them as combinations of cylinders [2]. Computational models, which emerged in the early 1960s, began to closely replicate the geometric intricacies of the human body and included realistic boundary conditions to simulate diverse environmental scenarios. These computational models, designed to mimic the entire human body, are commonly referred to as whole-body models [2]. The initial comprehensive model of the entire human body was developed by Professor Eugene Wissler's laboratory. This model, organized based on subdomains, represents distinct segments of the human body as 15 cylinders. Pennes' bioheat equation [1] was used to calculate the temperature distribution within each subdomain. Wissler's model took into account local physiological factors, such as variations in blood flow rate and metabolic heat generation. It also incorporated heat dissipation through the respiratory system and sweating. Each cylindrical subdomain featured a vascular network of countercurrent arteries and veins, allowing for the analysis of thermal exchange between tissue regions and blood. As a result, changes in body temperature could be calculated. The outcomes from Wissler's model closely matched experimental data [3], leading to its widespread adoption in the 1980s and 1990s for predicting changes in body temperature and blood temperature. Following Wissler's model, various other whole-body models were developed by researchers such as Smith [4], Fu [5], Fiala et al. [6], and Salloum et al. [7]. Similar to Wissler's methodology, these models integrated a vascular network into their structures. Zhu et al. [8] described the primary governing equations used in this study. Following the precedent of earlier whole-body models, Pennes' bioheat equation was applied in this research to predict temperature distribution within the human body, with arterial blood temperature serving as an input. The geometric representations of the human body in this investigation were simplified to basic shapes such as spheres or cylinders.

In contrast to prior studies, where vascular network analysis was replaced by an energy balance equation to assess changes in blood temperature during circulation, the recently developed whole-body model underwent testing by administering ice-slurry saline intravenously. This intervention aimed to counteract fever and reduce body-brain temperature following a head injury. The findings of the study [8] align closely with simplified energy balance calculations and clinical observations. This model shows promise for application in intricate thermoregulatory scenarios and dynamic changes in boundary conditions surrounding the human body. Accidental hypothermia refers to the involuntary drop of core temperature below 35 °C [9]. It can occur in a healthy individual due to exposure to a cold environment (primary accidental hypothermia) or be triggered by other conditions, commonly illness, intoxication, or trauma, termed secondary hypothermia. As accidental hypothermia progresses, vital signs decrease until cardiac arrest ensues. This condition has deleterious effects on the function of various organs, including the heart, brain, kidney, blood coagulation, and potentially the immune system [10,11]. Overall, accidental hypothermia heightens morbidity and mortality in affected individuals. Furthermore, in their research, Paul et al. [12] utilized a similar model to explore the effects of cold-water immersion at different temperatures, serving as inspiration for the development of our model. Their investigation employed a whole-body model incorporating tissue-blood interaction to simulate cooling during cold water immersion at varying temperatures (18.5 °C, 10 °C, and 0 °C). Survival time predictions were based on a 2–3 °C decrease in core body temperature corresponding to different water temperatures. While the study focused on the thermal responses of the human body during cold water immersion and exercise, it did not delve into the practical applications or implications of the findings. The primary computational output of whole-body models is the core body temperature, with the goal of maintaining close to the normothermic value of approximately 37 °C [13]. The circulatory system plays a pivotal role in redistributing blood perfusion throughout the body and adjusting cardiac output in response to cold or heat stress. A malfunction in the thermoregulatory system may result in severe physiological consequences, including organ failure and neurological deficits [14]. In a comprehensive review, Xu and Tikuisis [15] summarized advancements, achievements, and challenges within the domain of cold thermoregulatory modeling. Their study explored diverse applications, ranging from interpreting physiological observations to predicting outcomes such as skin freezing times and survival times in hypothermia. The research delved into the various mechanisms of heat exchange between the human body and cold environments, contributing to a comprehensive understanding of these interactions.

Cheshire [16] emphasized the importance of neural thermoregulatory mechanisms and the potential risks of extreme temperatures. Hypothermia, defined as a core temperature below 35.0 °C, can be life-threatening and requires warming measures, hydration, and cardiovascular support. Hyperthermia, on the other hand, necessitates immediate core temperature reduction, ideally through ice water immersion. However, there is a lack of sufficient information about the long-term consequences and complications of thermoregulatory disorders, which is a critical gap in developing comprehensive treatment strategies. The research on Thermal Balance and Survival Time Prediction of Individuals in Cold Water focuses on the effects of water temperature on metabolic heat production during immobilization [17]. This study reveals an inverse correlation between water temperature and the efficiency of the thermogenic response, which contributes to the development of hypothermia. Interestingly, swimming increases heat production but also accelerates cooling compared to remaining stationary, which has implications for predicting survival time. An equation that takes into account water temperature can be used for this purpose. Understanding these dynamics is essential for evaluating heat balance, cooling rates, and survival outcomes in situations involving immersion in cold water. Accidental hypothermia has been recognized since ancient times [18]. It was historically associated with warfare and catastrophic events such as avalanches, earthquakes, and tsunamis [[19], [20], [21]]. In developed countries, primary hypothermia primarily affects individuals who engage in outdoor activities or are homeless, exposing themselves to cold environments. In less developed nations, primary hypothermia impacts homeless individuals and victims of mass accidents, such as avalanches in mountainous areas that lack adequate protection. Generally, the risk of accidental hypothermia increases with decreasing temperatures, though instances among the homeless often occur during periods of low to moderate cold stress [22]. Secondary hypothermia is recognized, especially in elderly populations and those with multiple health issues, notably in Japan with its aging demographic [23]. Countries with similarly aging populations may witness comparable increases in the future. In the United States, primary hypothermia accounts for at least 1500 deaths annually. From 1995 to 2004, about 15,000 patients presented annually with hypothermia and cold-related conditions [24]. The incidence of accidental hypothermia in European countries and New Zealand varies from 0.13 to 6.9 cases per 100,000 per year [[25], [26], [27], [28]]. Scotland reports about 2 deaths per 100,000 per year, while Poland registers 5 per 100,000 per year [29,30]. The wide variation in reported frequencies is due to the absence of reliable national data for most countries, particularly in less developed regions like Africa, South America, and Southeast Asia. Different rewarming techniques exhibit varying rates. Passive rewarming ranges from 0.5 to 4 °C/h, depending on the patient's thermoregulatory function and metabolic reserves [3,31]. Passive rewarming with active movement can achieve a rate of 1–5 °C/h, but immediate exercise post-rescue may heighten the after-drop, potentially causing rescue collapse [32]. Active external rewarming, such as forced-air surface rewarming, offers controlled temperature increases of 0.5–4 °C/h [33,34]. Close monitoring of the patient's core temperature during rewarming is crucial to preventing complications such as after-drop and rewarming shock. Managing the rewarming rate carefully is essential to avoiding adverse effects and ensuring a safe and effective recovery for hypothermic patients. In this investigation, we utilize numerical simulations based on the finite element method and a realistic geometric model of the entire human body in COMSOL Multiphysics® [35]. The finite element analysis uses Penne's bio-heat equation [1]. Our model incorporates major heat transfer and thermoregulatory mechanisms, as well as circadian rhythm effects. To validate our model, we compare its temperature predictions with those of other models, such as Wissler's comprehensive whole-body model [3] and Paul's ANSYS Fluent whole-body model [12].

The focus of this study is to explore the complex interplay between survival durations, prehospital interventions, and scenarios involving cold-water immersion. We use computational modeling methods to shed light on the influence of varying water temperatures, water flow (both still and moving water), and water composition (pure water and saltwater) on individuals experiencing accidental cold-water immersion incidents. Our analysis reveals significant variations in survival times under different conditions of cold-water immersion. The study identifies the most effective active external rewarming fluid for the most critical hypothermal conditions. By quantifying the impact of these variables on survival times, the study provides data-driven recommendations to improve emergency protocols and outcomes for individuals facing cold-water immersion incidents.

## 2 Methods

### 2.1 Governing equation

Penne's bio-heat equation [1,36] stands as the predominant formula guiding the calculation of temperature distribution within human tissue. The distribution of heat in biological tissues is described by the Pennes bioheat equation, a mathematical model utilized in the study of bioheat transfer.

To analyze this governing equation in three dimensions, the weighted residual and domain decomposition methods were applied [37]. The Penne's bio-heat equations employed in COMSOL Multiphysics® are as follow [35]:

(1) $$\rho c_{p}\frac{\partial T}{\partial t}+\rho c_{p}u.\nabla T+\nabla .q=\rho_{b}c_{b}\omega_{b}\left(T_{b}-T\right)+Q_{m}+Q$$

(2) q=−k.∇T

Where, $c_{p}$ is the specific heat (J/kg °C), ρ is the density of the tissue (kg/m^3^), $\rho_{b}$ denotes the density of the blood (kg/m^3^), $c_{b}$ denotes the specific heat of the blood (J/kg °C), $\omega_{b}$ is the blood perfusion rate (1/s), $T_{b}$ is the known arterial temperature (°C), T is the unknown tissue temperature (°C), t is time (min), q is the heat flux ($W/m^{2}$), k is the thermal conductivity of the tissue (W/m °C), ${\;Q}_{m}$, is the volumetric metabolic heat (W/m^3^), and Q is the external heat source (W/m^3^).

### 2.2 Flow of analysis

A realistic human body model was created by integrating geometric shapes to represent various body components, including limbs, torso, neck, internal organs, and head. A skin layer was added to simulate vasoconstriction effects in different situations, enhancing the overall realism [3]. The entire process, which involved establishing boundary conditions, generating meshes, solving equations, and visualizing results, was carried out using COMSOL Multiphysics® [35]. The graphs were plotted in OriginPro 2024 [38], with the data extracted from COMSOL Multiphysics®.

## 3 Model geometry and materials

### 3.1 Model geometry

**Fig. 1:**
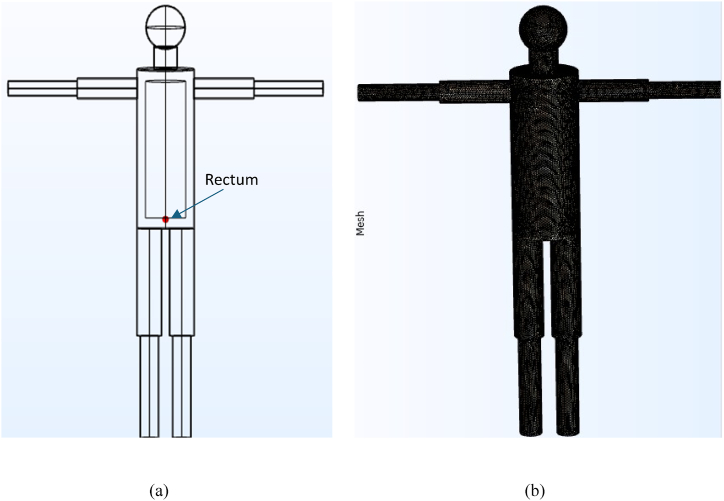
(a) The geometry of a realistic human model and (b) the finite element mesh of the model.

This investigation utilizes a human body model (Fig. 1) specifically designed for research purposes. Fig. 1(a) shows the structure of a realistic 3D human body model, including limbs, torso, neck, internal organs, head, and skin. Fig. 1(b) displays the model's finite element mesh.

To simulate vasoconstriction effects [3], a 4 mm layer of skin [36,39] was integrated into the model. Kuwabara et al. [40] aimed to develop an equation for estimating the convective heat transfer coefficient in humans, particularly in outdoor settings. Their findings indicated that the coefficient for convective heat transfer in both unclothed and clothed individuals was nearly identical. The anthropometric details of the model included a height of 1.684 m, a calculated body surface area (BSA) of 1.7 m^2^, and a body volume of 0.057 m³. The skin surface area of the whole body was measured by summing all the cylindrical and spherical surface areas exposed to the surroundings. The volume of the body was measured by summing the volumes of cylinders exposed to the environment. The average density of the body is 1060 kg/m³ [8,12]. The weight of the body is 60.5 kg (volume * density), and the BMI is 21.34 (weight/${\text{height}}^{2}$) [41]. These dimensions were selected to match the average size of individuals from the South Asian Subcontinent, such as Bangladesh and India. The computational analysis of the entire body model was carried out using the finite element solver, COMSOL Multiphysics®. A mesh independence study was conducted to ensure accuracy, refining the finite element mesh until a stable state in the change of steady state was reached. The 3D design employed in this analysis consisted of 7,489,689 quadratic tetrahedral elements, and 1,308,837 vertices.

### 3.2 Physiological properties

Similar to the model proposed by Zhu et al. [8,12], the main computational components include the muscle, head, internal organs, and skin. Table 1 presents a concise overview of the physical and physiological attributes of these elements under standard environmental conditions. The table outlines the key parameters for the entire-body thermal model.

**Table 1:** Physiological properties of tissues [8,12,42].

Tissues	Thermal conductivity (W/m°C)	Density $\left(kg/m^{3}\right)$	Specific heat (J/kg°C)	Blood perfusion (1/s)	Metabolic heat generation $\left(W/{m\;}^{3}\right)$
Muscle	0.5	1060	3800	0.0005	553.5
Skin	0.3	1060	2802	0.0005	553.5
Head	0.5	1060	3800	0.00833	9225
Internal organs	0.5	1060	3800	0.001266	1401.5

### 3.3 Boundary condition

To analyze a temporary heat transfer phenomenon, it is important to define the initial conditions. In this study, we assume that the initial state is normothermic, meaning that there is a stable temperature distribution within the model. This equilibrium occurs when the body reaches a thermal balance with its surroundings. The internal temperature of the body, including the muscles, internal organs, and the head, is set at 37 °C [36], which is the typical initial temperature of human muscle. Similarly, the initial mean temperature of the skin is established at 34.5 °C [15]. These initial temperatures may vary depending on the temperature and type of fluid in which the human body is immersed. The main methods of heat transfer from the human body to the surrounding fluids are convection, radiation, and evaporation. However, it is important to note that radiation and convection are significant in air, not in water. In our investigation, we combine the convection and radiation mechanisms into an overall heat transfer coefficient (h) as part of the specified boundary conditions for immersion in water. We assume that evaporation is negligible in this case. The equation used for the boundary condition in COMSOL Multiphysics® is as follows [35]:

(3) $$q_{o}=h\;\left(T_{ext}-T\right)$$

Where, $q_{o}$ is heat flux form human body to the surroundings (W/m °C), h is the overall heat transfer coefficient (W/m^2^ °C), T is unknown tissue temperature (°C), and $T_{ext}$ is the environmental temperature (°C).

## 4 Results and discussions

### 4.1 Model Validation-1

**Fig. 2:**
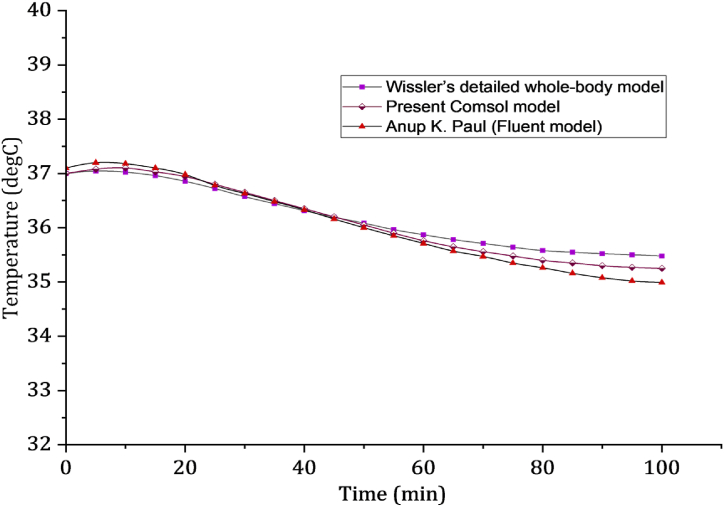
Presents the body core temperature profiles of various whole-body models while being immersed in still water at 18.5 °C.

Fig. 2 presents a visual representation of the calculated fluctuations in core body temperature during the immersion of the human body in still water at 18.5 °C, with an overall heat transfer coefficient of 139 $W/m^{2}{}^{\circ}C$ [12]. This analysis takes into account the heat generated by shivering. By considering both the skin layer and the impact of vasoconstriction, the computed temperature exhibits a gradual decline and eventually stabilizes. It is worth noting that the computed temperature from our computational model, which incorporates vasoconstriction in the skin layer, closely aligns with the results obtained from Wissler's comprehensive whole-body model [3] and Paul's ANSYS Fluent whole-body model [12], as shown in Fig. 2. Our model differs by 1.3 % and 1.5 % from Wissler's comprehensive whole-body model and Paul's ANSYS Fluent whole-body model, respectively. COMSOL Multiphysics® and ANSYS might yield different results due to variations in their algorithms, element types, meshing techniques, and solver implementations however the difference was very little (1.5 %).

### 4.2 Water temperatures, survival times, and cooling rates

Hypothermia occurs when the body temperature drops below 35 °C. It can happen after prolonged exposure to temperatures below 10 °C or extended immersion in cold water below 20 °C. When the body temperature falls below 32 °C, it becomes a life-threatening condition [43]. Shivering is a bodily response that usually stops when the body temperature drops below 90 °F (32 °C) [44]. Cardiac arrhythmia and death can occur when the body temperature is below 30 °C [45].

Hayward et al. [17] experimentally developed equations to predict rectal temperature $\left(T_{R}\right)$ a function of immersion time ($t_{s}$ in minutes) and water temperature ($T_{w}$):

(4) $$T_{R}=37.2\;-\;\left(t_{s}-\;15\right)\left(0.0785\;-\;0.0034T_{w}\right)$$

when $t_{s}$ ≥ 15 min and $T_{w}$ ≤ 23 °C.

A prediction equation for surviva1 time **(**$t_{s}$) of persons accidently immersed in cold water ($T_{w}$) has the form [17]:

(5) $$t_{s}=15+\frac{\;7.2}{\left(0.0785\;-\;0.0034T_{w}\right)}$$

in which $t_{s}$ is survival time in minutes, $T_{w}$ is the water temperature in degrees Celsius (≤23 °C), and “incipient” death is assumed to occur at a rectal temperature of 30.0 °C**.**

Cooling rate (C) due to cold water [17]:

(6) $$C=\left(0.0785\;-\;0.0034{\;T}_{w}\right)$$

Using Eqs. (4), (5), (6) the rectal temperatures, the survival tines, and the cooling rates for different temperatures of water are calculated, which are provided in Table 2.

**Table 2:** Rectal temperatures, survival times, and cooling rates for different temperatures of water [17].

Water temperature, $T_{w}$ (°C)	Surviva1 time, $t_{s}$ (min)	Rectal temperature, $T_{R}$ (°C)	Cooling rate, C (°C/min)
5	132	30	0.0615
2	115	30	0.0717
0	106	30	0.0785

### 4.3 Model Validation-2

The survival times of the present model have been validated through experimental work conducted during steady cold-water immersion. Section 4.4 explains the survival times at different water temperatures. It is worth noting that the findings of this study closely align with the results obtained by Hayward et al. [17], which are summarized in Table 3. The research also nearly confirms the findings from the experimental investigations by Molnar [46] and Hayward et al. [47] on water at 5 °C, as shown in Table 4. There are slight differences between the experimentally predicted results and the present numerical results because an average human body model was used in the present model. This means that individuals with different heights, weights, and ages were not included.

**Table 3:** Survival times for different temperatures of water.

Water temperature, $T_{w}$ (°C)	Surviva1 time, $t_{s}$ (min)		
	Hayward et al. (1975)	Present study	Deviation (%)
5	132	136	3 %
2	115	113	1.7 %
0	106	100	5.6 %

**Table 4:** Survival times observed in experimental studies and present study.

Water temperature, $T_{w}$ (°C)	Surviva1 time, $t_{s}$ (min)		
	Present study	Molnar [46]	Hayward et al. [47]
5	136	132	138

### 4.4 Duration of survival in accidental coldwater immersion

The heat transfer coefficient (h) is influenced by various factors such as the properties of the fluid (density, viscosity, thermal conductivity), flow characteristics, and surface conditions. Generally, saltwater has a higher heat transfer coefficient compared to pure water due to its higher thermal conductivity resulting from the presence of dissolved salts. The numerical values of heat transfer coefficients when a human body is immersed in running cold saltwater and cold pure water depend on several factors, including the exact temperature differentials, flow rates, and the specific composition of the fluids. However, we can provide some general ranges based on typical conditions.

In cold freshwater, with temperatures ranging from 0 °C to 10 °C, the heat transfer coefficient can fall within the higher end of the spectrum, approximately 100–230 $W/m^{2}{}^{\circ}C$ [15]. This range is subject to variations influenced by factors like water temperature and body surface area, with our study adopting a value of 220 $W/m^{2}{}^{\circ}C$. In the context of dynamic immersion, such as swimming in freshwater, the heat transfer coefficient experiences a notable increase due to enhanced convective heat transfer. This coefficient may vary between 427 and 580 $W/m^{2}{}^{\circ}C$ at water velocities of 0.5–0.75 m/s [15], and for our research, a value of 460 $W/m^{2}{}^{\circ}C$ has been applied.

When considering static immersion in saltwater, the heat transfer coefficient might be slightly higher compared to fresh water due to the lower thermal conductivity of pure water. The heat transfer coefficient in cold saltwater can range from approximately 100 to 500 $W/m^{2}{}^{\circ}C$ under typical conditions [48,49]. However, our study employs a specific value of 480 $W/m^{2}{}^{\circ}C$. In the case of dynamic immersion in cold saltwater with similar temperature ranges, the coefficient could range from around 500 to 1000 $W/m^{2}{}^{\circ}C$ [[50], [51], [52]], and our study utilizes a value of 680 $W/m^{2}{}^{\circ}C$. It is noteworthy that this coefficient may decrease when the water temperature approaches body temperature, resulting in a reduced temperature gradient for heat transfer. The correlation between skin temperature and sensation varies above and below thermal neutrality. Below the neutral point, a decline in skin temperature occurs with a decreasing ambient temperature. As the temperature drops below 33.5 °C (92 °F), corresponding to a comfortable level, the perception of cold sensations intensifies, contributing to a gradual increase in cold discomfort [53]. This relationship is estimated by computing the weighted sum of skin temperatures across different parts of the body [53]:

(7) $$T_{s}=0.12T_{back}+0.12T_{chest}+0.12T_{abdomen}+0.14T_{arm}+0.19T_{thigh}+0.13T_{leg}+0.05T_{hand}+0.07T_{head}+0.06T_{foot}$$

Assessing the perception of cold sensation involves measuring skin temperature, a method outlined in ISO 11079 [54]. ISO 11079 states that heightened physiological strain associated with feeling “cold” involves peripheral vasoconstriction and the absence of regulatory sweating. The average skin temperature linked to the sensation of “cold” is influenced by the metabolic rate. In the peripheral regions, the initial perception of skin cooling begins at around 28 °C, with pain sensed at 20 °C and numbness occurring at 0 °C or below [55]. When the body temperature falls below 32 °C, it becomes a life-threatening condition [43].

Cardiac arrhythmia and death may occur when the body temperature is < 30 °C [45]. Hayward et al. [17] propose that when the human body is immersed in cold water with a temperature ($T_{w}$) equal to or below 23 °C, “incipient” death is assumed to occur when the rectal temperature reaches 30.0 °C.

**Fig. 3:**
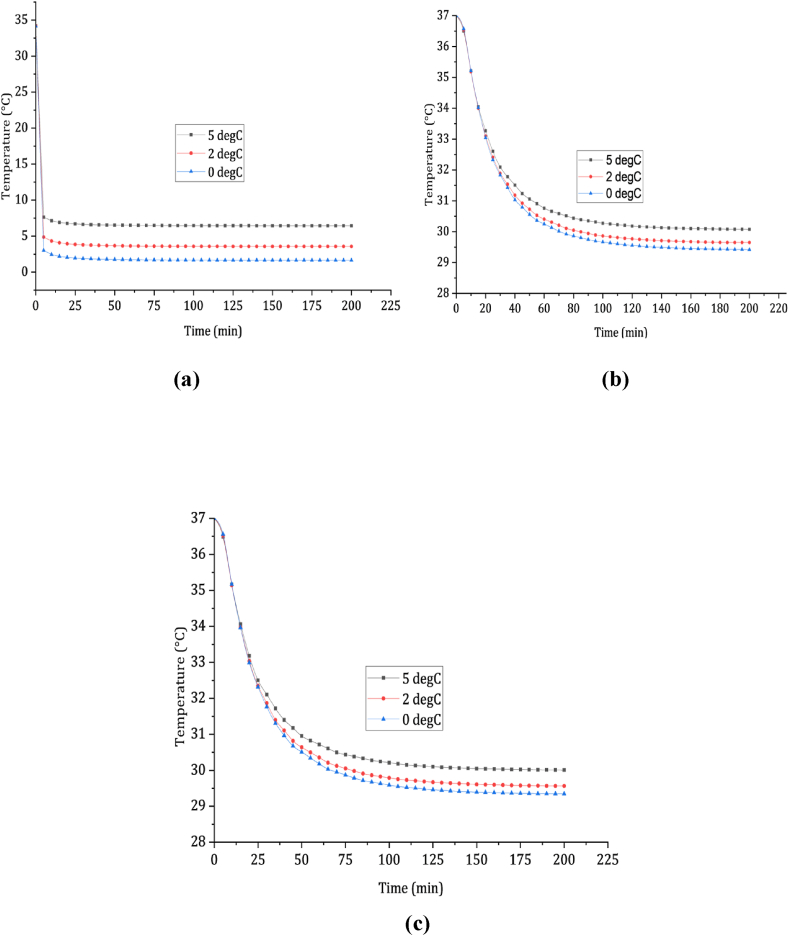
Illustrates the cooling profiles of the skin and rectum during both still and moving cold-water immersion. In (a) and (b), we see the cooling profiles of the skin and rectum, respectively, in still cold-water immersion. In (c), we observe the rectum cooling profiles specifically in moving cold-water immersion.

Fig. 3(a) and (b) depict temperature profiles of the skin and rectum during immersion in steadily cold water at different temperatures (5, 2, and 0 °C). Fig. 3(a) illustrates a rapid decline in skin temperature within the first 5 min, stabilizing after 25 min. Skin temperature, following immersion in 5, 2, and 0 °C water, drops from 34.5 to 7.63, 4.86, and 3.04 °C, respectively. Beyond 25 min, the skin temperature approaches the water temperature, becoming nearly constant. The rapid drop in skin temperature below 20 °C within 1-min causes thermal shock when immersed in water below 5 °C, leading to immediate pain.

Fig. 3(b) displays rectal temperature profiles over time, indicating a significant decrease in the first 40 min during immersion in water at temperatures of 5, 2, and 0 °C. The average cooling rates for these temperatures are 0.0533, 0.068, and 0.0805 °C/min, respectively. Rectal temperature reaches 30 °C after 136, 113, and 100 min of immersion in 5, 2, and 0 °C water. Therefore, based on Hayward et al. [17], the estimated survival times for an average person in steady water with temperatures of 5, 2, and 0 °C are 136, 113, and 100 min, respectively.

In Fig. 3(c), the rectal temperature profiles depict a rapid decrease in temperature during the initial 50 min when immersed in flowing water at 5, 2, and 0 °C. The average cooling rates for these three water temperatures are 0.0585, 0.079, and 0.0986 °C/h, respectively. The rectal temperature reaches 30 °C after 119, 92, and 83 min of immersion in water at 5, 2, and 0 °C. Consequently, the survival times for an average individual in running water at these temperatures are 119, 92, and 83 min, respectively.

It is noteworthy that the survival time in running cold water is shorter compared to steady cold water. On average, the survival time during immersion in running cold water is approximately 16 % less than that in steady cold-water immersion.

### 4.5 Survival time in cold saltwater accidental immersion

The freezing point of seawater is generally lower than that of freshwater due to the presence of dissolved salts. Typically, seawater freezes at approximately −2 °C (28.4 °F), with variations influenced by factors such as salinity and pressure [56].

Vulnerable or poorly insulated parts of the body, such as extremities and facial protrusions like ears and noses, are more susceptible to frostbite. Frostbite involves tissue freezing, commencing at the skin surface, with a freezing point of around −0.6 °C, as initially documented by Keatinge [57].

**Fig. 4:**
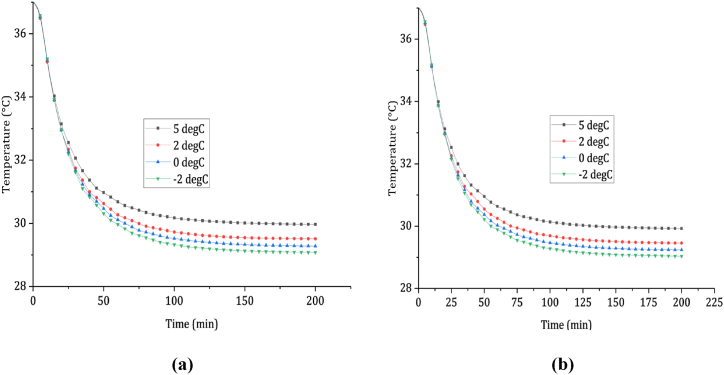
Shows the cooling profiles of the rectum in (a) still-cold saltwater immersion and (b) dynamic cold saltwater immersion.

In Fig. 4 (a), the rectal temperature profiles depict a rapid decrease in temperature during the first 45 min of immersion in still saltwater at temperatures of 5, 2, 0, and −2 °C. The average cooling rates for these temperatures are 0.0594, 0.083, 0.1, and 0.115 °C/min, respectively. The rectal temperature reaches 30 °C after 111, 88, 80, and 66 min of steady saltwater immersion at temperatures of 5, 2, 0, and −2 °C. Hence, the survival times for an average person in steady saltwater at temperatures of 5, 2, 0, and −2 °C are 111, 88, 80, and 66 min, respectively.

In Fig. 4 (b), the rectal temperature profiles indicate a rapid decrease in temperature during the first 55 min of immersion in running saltwater at temperatures of 5, 2, and 0 °C. The average cooling rates for these temperatures are 0.0648, 0.0986, 0.113, and 0.135 °C/min, respectively. The rectal temperature reaches 30 °C after 98, 74, 68, and 57 min of running saltwater immersion at temperatures of 5, 2, 0, and −2 °C.

Consequently, the average survival times for a person in saltwater at temperatures of 5, 2, 0, and −2 °C are 98, 74, 68, and 57 min, respectively. When comparing survival times, it is found that the duration is shorter in running cold water compared to steady cold water. On average, the survival time is approximately 13.4 % shorter in running cold saltwater immersion than in steady cold saltwater immersion.

### 4.6 Cooling effects of fresh water and saltwater

**Fig. 5:**
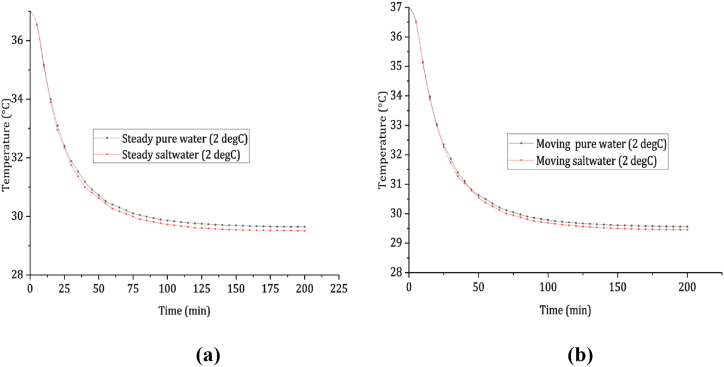
Showcases the cooling profiles of the rectum, comparing two different scenarios: (a) immersion in still cold fresh water and saltwater, and (b) immersion in dynamic cold fresh water and saltwater.

The impact of water temperature on cooling effects was examined by analyzing a specific scenario involving a water temperature of 2 °C. This analysis aimed to compare the cooling properties of pure water and saltwater. Based on the data presented in Fig. 5(a) and (b), the average cooling rates of the rectum were found to be 0.064 and 0.083 °C/min for steady immersion in pure water and saltwater, respectively. For moving conditions, the average cooling rates of the rectum were 0.0786 and 0.1 °C/min for pure water and saltwater immersion, respectively. As a result, the survival times for an average individual were calculated to be 113 and 88 min when immersed in steady pure water and saltwater at 2 °C, and 92 and 74 min for immersion in moving conditions. Comparing the outcomes for both pure water and saltwater, it was observed that the average survival time for pure water immersion exceeded that of saltwater by 22.7 % under steady conditions and 22.2 % under moving conditions.

### 4.7 Causes behind the demise of Titanic's passengers

**Fig. 6:**
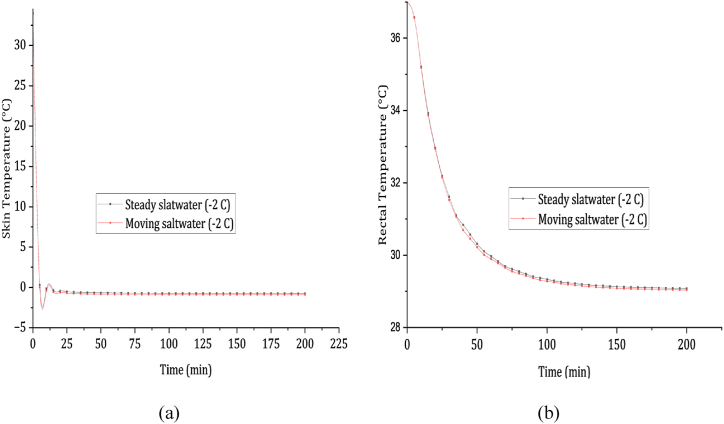
(a) Skin cooling profile and (b) Rectum cooling profile in still and dynamic cold saltwater immersion.

The tragic sinking of the Titanic took place in the North Atlantic Ocean on April 15, 1912, with the water temperature at approximately −2 °C (28 °F) [58]. The freezing conditions of the water greatly contributed to the disaster, reducing the chances of survival for those thrown into the sea [58]. The duration of survival varied depending on factors such as age, health, and proximity to lifeboats. Many passengers and crew members succumbed to hypothermia within minutes of being immersed in the icy waters. Estimates suggest a survival window of about 15–30 min before succumbing to the harmful effects of cold shock and hypothermia [59]. Contrary to popular belief, sudden exposure to freezing water usually leads to rapid death, caused by cardiac arrest, involuntary inhalation of water, or cold shock, rather than hypothermia [60]. Frostbite, the freezing of the skin, nerves, and blood vessels under the skin's surface due to extreme cold, occurs when rain, snow, water, or wind accelerate skin cooling. Wet skin and clothing worsen heat loss, potentially resulting in frostbite. The freezing point for tissue begins at about −0.6 °C, as originally reported by Keatinge [57]. Fig. 6 (a) illustrates the skin temperature dropping from 34.5 to −1 °C within the first 5 min of immersion in steady and moving saltwater at −2 °C. After 25 min, the skin temperature stabilizes, approaching the water temperature. The rapid decline, reaching below −0.6 °C within a minute, exposes the human body to severe thermal shock. In Fig. 6 (b), survival times for an average person in steady saltwater and moving saltwater at −2 °C are 66 and 57 min, respectively. However, the majority of Titanic passengers and crew members perished within 15–30 min [59].

The fatalities predominantly occurred within a 15–30 min timeframe. Our analysis indicates that these deaths were caused by intense thermal shock rather than hypothermia. If hypothermia were the cause, individuals could have potentially survived for at least 51 min. Therefore, based on this data, our conclusions suggest that the main factor leading to their demise was severe thermal shock rather than hypothermia.

### 4.8 Prehospital hypothermia treatment

Various methods of rewarming exhibit different rates of rewarming, and the selection of a specific technique hinges on the patient's condition and available resources. The pace of rewarming is a crucial aspect of managing hypothermic patients. This discussion outlines several common rewarming techniques and their associated rates:

Passive rewarming typically progresses at a rate of 0.5–4 °C/h, influenced by the patient's thermoregulatory function and metabolic reserves [3,31]. When combined with active movement, passive rewarming can achieve a faster rate of 1–5 °C/h; however, immediate exercise post-rescue may elevate the risk of after-drop, potentially resulting in rescue collapse [32]. Active external rewarming, specifically forced-air surface rewarming (0.5–4 °C/h), provides controlled core temperature elevation [33,34]. Extra-corporeal life support (ECLS; VA-ECMO; CPB, including minimally invasive extracorporeal circulation (MiECC)), with a rewarming rate of ∼4–10 °C/h, is the preferred method for cardiac arrest patients, with ECMO being favored over CPB [[61], [62], [63], [64], [65], [66], [67], [68], [69], [70], [71], [72]].

Close monitoring of the patient's core temperature during rewarming is essential to prevent complications such as after-drop and rewarming shock. Careful management of the rewarming rate is crucial to ensuring a safe recovery for hypothermic patients. Swift rewarming in severe cases may lead to complications like rewarming shock or an after-drop, where cold blood returning from extremities causes a drop in core temperature, potentially triggering adverse reactions [73].

This study focuses on active external rewarming methods, including whole-body immersion in forced Lukewarm air, Lukewarm still water and moving water. Determining the heat transfer coefficient for a human body in lukewarm forced air involves considering factors like air temperature, velocity, humidity, and body surface area, with typical values ranging from 5 to 25 $W/m^{2}{}^{\circ}C$ [48,74].

The heat transfer coefficient in lukewarm water is influenced by factors such as water temperature, body composition, and immersion duration, with a commonly cited range of approximately 100–235 $W/m^{2}{}^{\circ}C$ [15,75]. In this study, a value of 220 $W/m^{2}{}^{\circ}C$ is utilized. Similarly, for lukewarm moving water, the coefficient ranges from 427 to 580 $W/m^{2}{}^{\circ}C$ at water velocities of 0.5–0.75 m/s [15,75], with this study adopting a value of 480 $W/m^{2}{}^{\circ}C$.

**Fig. 7:**
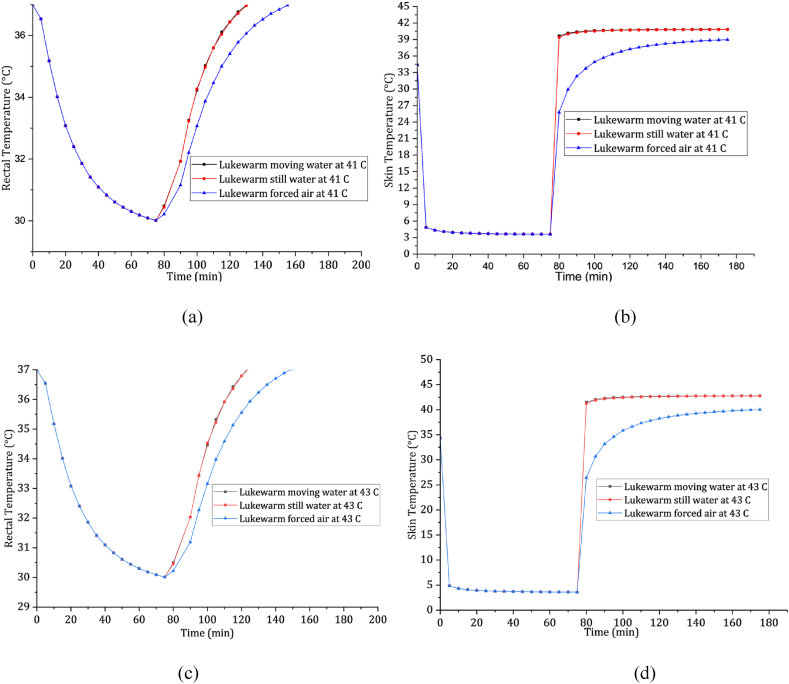
Rewarming profile in lukewarm waters and lukewarm forced air. Panels (a) and (c) represent rectum rewarming at 41 °C and 43 °C, respectively. Panels (b) and (d) depict skin rewarming at 41 °C and 43 °C, respectively.

Fig. 7 (a), (b), (c), & (d) illustrate the rewarming rates for different treatments. These figures show that the rectal temperature returns to 37 °C after approximately 60 min for all water types at 41 °C, with a rewarming rate of 7 °C/h. Lukewarm forced air at 41 °C exhibits a rewarming rate of 4.9 °C/h. Similar patterns are observed at a lukewarm temperature of 43 °C, with the rectal temperature returning to 37 °C after about 50 min and a rewarming rate of 8.4 °C/h for water treatments. Lukewarm forced air at 43 °C has a rewarming rate of 5.6 °C/h. To prevent tissue damage, it is crucial to keep the rewarming fluid temperatures within the 43 °C threshold, as temperatures exceeding this threshold are associated with tissue damage [76]. Immersion in lukewarm water (41–43 °C) emerges as the most effective prehospital treatment for severe hypothermia. It helps maintain the skin temperature below the critical threshold of 43 °C, potentially saving lives before conventional medical interventions.

## 5 Assumptions and limitations

Some assumptions have been made in this study, including the use of simplistic tissue models and assumptions about tissue uniformity. To conduct this study, an average human body model was used, which means that individuals with different heights, weights, and ages were not included. As a result, there is a lack of sensitivity analysis. The convection and radiation mechanisms have been combined into an overall heat transfer coefficient (h) as part of the specified boundary conditions. Additionally, evaporation has been assumed to be zero for immersion in water. Furthermore, the heat transfer coefficients are considered to be the same for all temperatures of the cold water. Although it is possible for the coefficient to vary with temperature, we have assumed it to be constant due to the lack of specific values for different temperatures in existing literature. Because of the swift increase in temperature during the rewarming process, the body may experience thermal stress and develop cracks, leading to various hazardous outcomes. However, it is important to note that this study did not specifically investigate these conditions.

## 6 Conclusions

This study uses computational modeling to investigate the complex relationship between scenarios of cold-water immersion, survival durations, and prehospital interventions. By examining different water temperatures, including fresh and salt water, the research reveals significant differences in survival outcomes during accidental cold-water incidents. For example, individuals immersed in water at temperatures of 5 °C, 2 °C, and 0 °C experienced average survival times of 136, 113, and 100 min, respectively, under steady conditions. In flowing water at the same temperatures, survival times decreased to 119, 92, and 83 min. Importantly, individuals submerged in 5 °C, 2 °C, and 0 °C water demonstrated shorter average survival times in running cold water compared to steady conditions, with an approximately 16 % reduction in survival time. This indicates the impact of water movement on cooling rates and survival durations. Similarly, individuals immersed in saltwater at temperatures of 5 °C, 2 °C, 0 °C, and −2 °C had average survival times of 111, 88, 80, and 66 min, respectively, in static conditions. In flowing saltwater at the same temperatures, survival times decreased to 98, 74, 68, and 57 min. Likewise, individuals in saltwater at 5 °C, 2 °C, 0 °C, and −2 °C experienced lower average survival times in running cold saltwater, showing a 14.4 % decrease compared to steady conditions. This emphasizes the impact of water flow on cooling rates and survival durations. Comparing pure water and saltwater immersion at 2 °C revealed survival times of 113 and 88 min in stable conditions and 92 and 74 min in dynamic conditions. This comparison shows a 22.7 % and 22.2 % higher average survival time for pure water in steady and moving conditions, respectively, highlighting the role of water composition. The study also challenges the belief that the demise of the Titanic's passengers and crew was caused by hypothermia, instead arguing that severe thermal shock was the primary cause. These numerical findings underscore the importance of considering water temperature, flow dynamics, and prompt medical responses in cold-water emergencies to enhance survival prospects. The study identifies water within the range of 41–43 °C as the most effective active external rewarming fluid for critical hypothermal conditions. By quantifying the impact of these variables on survival times, the study provides data-driven recommendations to improve emergency protocols and outcomes for individuals facing cold-water immersion incidents.

## Ethical approval

No experiments involving human tissue were carried out, so no ethical approval required by an institutional review board or equivalent ethics committee.

## Funding

This research did not receive any specific grant from funding agencies in the public, commercial, or not-for-profit sectors.

## Data availability

Data will be made available on request.

**Table:** Nomenclature

$\omega_{b}$	blood perfusion rate (1/s)
$T_{c}$	body core temperature (°C)
$C_{r}$	cooling rate (°C/h)
$\rho_{b}$	density of blood $\left(\text{kg}/m^{3}\right)$
ρ	density of the tissue $\left(\text{kg}/m^{3}\right)$
$T_{\alpha }$	fluid temperature (°C)
h	heat transfer coefficient ($W/m^{2\;}{}^{\circ}C$)
$T_{b}$	known blood temperature (°C)
$Q_{m}$	metabolic heat generation rate ($W/m^{3}$)
C	specific heat (J/kg°C)
$C_{b}$	specific heat of blood (J/kg°C)
$t_{s}$	Survival time (min)
k	thermal conductivity of the tissue (W/m°C)
t	time (s)
T	unknown tissue temperature (°C)
$T_{w}$	Water temperature (°C)

## CRediT authorship contribution statement

**Mohammad Junaid:** Writing – review & editing, Writing – original draft, Visualization, Validation, Supervision, Resources, Project administration, Methodology, Investigation, Formal analysis, Data curation, Conceptualization. **Md Mahmud-Or-Rashid:** Writing – review & editing, Investigation.

## Declaration of competing interest

The authors declare that they have no known competing financial interests or personal relationships that could have appeared to influence the work reported in this paper.
